# Establishment of coverage-mass equation to quantify the corrosion inhomogeneity and examination of medium effects on iron corrosion

**DOI:** 10.1093/rb/rbad007

**Published:** 2023-02-01

**Authors:** Xin Li, Jiandong Ding

**Affiliations:** State Key Laboratory of Molecular Engineering of Polymers, Department of Macromolecular Science, Fudan University, Shanghai 200438, China; State Key Laboratory of Molecular Engineering of Polymers, Department of Macromolecular Science, Fudan University, Shanghai 200438, China

**Keywords:** biomaterials, biodegradable metal, corrosion inhomogeneity, coverage-mass equation, biomimetic media, biodegradable medical device

## Abstract

Metal corrosion is important in the fields of biomedicine as well as construction and transportation etc. While most corrosion occurs inhomogeneously, there is so far no satisfactory parameter to characterize corrosion inhomogeneity. Herein, we employ the Poisson raindrop question to model the corrosion process and derive an equation to relate corrosion coverage and corrosion mass. The resultant equation is named coverage-mass equation, abbreviated as C-M equation. We also suggest corrosion mass at 50% coverage, termed as half-coverage mass *M*_corro50%_, as an inhomogeneity parameter to quantify corrosion inhomogeneity. The equation is confirmed and the half-coverage mass *M*_corro50%_ is justified in our experiments of iron corrosion in five aqueous media, normal saline, phosphate-buffered saline, Hank’s solution, deionized water and artificial seawater, where the former three ones are biomimetic and very important in studies of biomedical materials. The half-coverage mass *M*_corro50%_ is proved to be more comprehensive and mathematically convergent than the traditional pitting factor. Iron corrosion is detected using visual observation, scanning electron microscopy with a build-in energy dispersive spectrometer, inductive coupled plasma emission spectrometry and electrochemical measurements. Both rates and inhomogeneity extents of iron corrosion are compared among the five aqueous media. The factors underlying the medium effects on corrosion rate and inhomogeneity are discussed and interpreted. Corrosion rates of iron in the five media differ about 7-fold, and half-coverage mass values differ about 300 000-fold. The fastest corrosion and the most significant inhomogeneity occur both in biomimetic media, but not the same one. The new equation (C-M equation) and the new quantity (half-coverage mass) are stimulating for dealing with a dynamic and stochastic process with global inhomogeneity including but not limited to metal corrosion. The findings are particularly meaningful for research and development of next-generation biodegradable materials.

## Graphical Abstract

**Figure:**
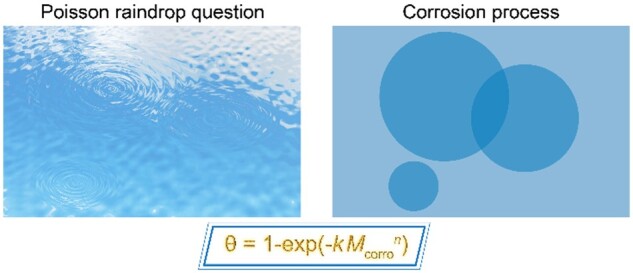

## Introduction

While metal is an important material type in science, industry and human life, its corrosion is a classic problem and has caused about 3% loss of the gross domestic product globally [1–3]. The destruction of integrity of a material due to corrosion can lead to unexpected disaster and casualties [4, 5]. According to Web of Science, there is about 580 000 ‘corrosion’ pertinent publications during the latest decade and the study of corrosion is increasing annually, as summarized in Supplementary Fig. S1. What is more, corrodible metals have been tried as the next-generation biodegradable materials [6], and it is much required to fundamentally investigate the corrosion behavior in biomimetic media.

In the research of metal corrosion, scientists focus mostly on corrosion rate and corrosion morphology in evaluation of the corrosion process [7, 8]. In fact, the corrosion of metal frequently occurs through pitting corrosion, crevice corrosion and so on, anyway heterogeneously or inhomogeneously [9–12]. However, there is so far no satisfactory parameter to evaluate the corrosion inhomogeneity in a unified way.

**Figure 1.**
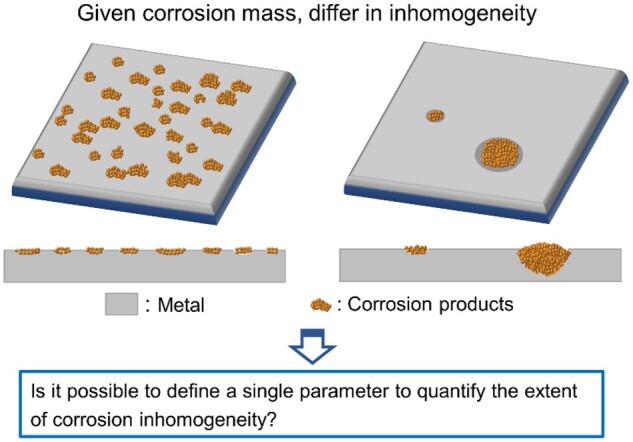
Schematic presentation of corrosion inhomogeneity.

As a key aspect to the corrosion process, the corrosion inhomogeneity is as important as corrosion rate. While corrosion rate can be quantified using a single parameter, corrosion inhomogeneity is described usually from several aspects. It has been challenging to quantify the extent of corrosion inhomogeneity, and the conventional ‘pitting factor’ about metal corrosion is not satisfactory, which will be discussed later. Herein, we schematically present this classic problem in Fig. 1. To solve this problem about corrosion inhomogeneity, we have employed the Poisson raindrop question, which was addressed by a French mathematician Poisson about 200 years ago in dealing with a statistical problem [13].

In the present work, a new equation is derived by us to relate corrosion coverage to corrosion mass, and a parameter is suggested by us to quantify the corrosion inhomogeneity. This article is aimed to report this coverage-mass (C-M) equation and the corrosion inhomogeneity quantity for the first time. High corrosion inhomogeneity of iron has been observed in previous publications from our group and other groups [14, 15]. The inhomogeneous corrosion may affect the mechanical strength of a medical device and thus affect its safety and performance. Our theoretical work was confirmed in experiments of iron corrosion in normal saline (NS) with NaCl and physiological osmotic pressure, phosphate-buffered saline (PBS) to mimic tissue fluid and Hank’s solution (HS) to mimic blood plasma. We also set deionized water (DI) as a control, and artificial seawater (AS) as the extension to enhance the potential impact of our work.

Scanning electron microscopy (SEM) and inductive coupled plasma emission spectrometry (ICP) were used to acquire corrosion images and corrosion mass. Fitting of the experimental data by C-M equation led to the mass loss at 50% corrosion coverage, which is defined by us as *M*_corro50%_. This new parameter was found to well reflect the corrosion inhomogeneity. What is more, the corrosion inhomogeneity was illustrated to be independent from corrosion rate.

## Materials and methods

### Preparation of samples

Pure iron foil with the purity of 99.99% was chosen for the study of corrosion behaviors in our work. The iron sheet or foil with the thickness of 200 μm was cut into the size of 10 mm (length) × 10 mm (width) for preparation of the specimens. The epoxy resin was used to seal the edge and back of the cut iron foil, and only one surface was exposed to the immersion medium. The iron surface was polished by sandpapers (SiC) of P800, P1500, P2000 and P3000 grits successively and then ultrasonically cleaned with ethanol and acetone for three times. Finally, the polished specimens were blown by nitrogen gas and dried in oven at 60°C.

### Immersion tests of iron in different media

Our immersion experiments were carried out in five media including DI, AS, NS, PBS and HS. Except DI, the other four media were prepared with inorganic salts and, in the case of HS, also glucose. As the simplest biomimetic solution, NS contained 9 g NaCl per liter. The AS contained 23.56 g NaCl, 6.6 g MgCl_2_, 0.7 g KCl, 0.2 g NaHCO_3_, 1.56 g CaCl_2_ and 4.01 g Na_2_SO_4_ per liter. For the preparation of PBS, a commercial PBS powder was used with the concentration of 0.01 M phosphate salt, and the solution had the balanced pH of 7.4. HS contained 8.0 g NaCl, 0.4 g KCl, 0.35 g NaHCO_3_, 0.1 g MgSO_4_⋅7H_2_O, 0.1 g MgCl_2_⋅6H_2_O, 0.012 g NaH_2_PO_4_⋅12H_2_O, 0.06 g K_2_HPO_4_, 0.14 g CaCl_2_ and 1.0 g glucose in per liter. After preparation of the solutions, the pH of media was adjusted to 7.4 with diluted aqueous solutions of HCl and NaOH except for DI before specimen immersion. The iron specimens were hung and immersed in 500 ml media. The static immersion conducts in a water bath at 37°C. The media were changed every 48 h to maintain the static pH and dissolved oxygen.

### Observation of the morphology of corroded specimens

The iron sheets after immersion in different media were globally observed with a digital camera. SEM in a microscope (LaB6-SEM, VEGA 3 XMU, TESCAN, Germany) was used to observe the surface morphologies with and without removal of corrosion products. Corrosion products were removed using a hydrogen chloride solution with 3.5 g hexamethylenetetramine per liter. For the preparation of the cross-sections, the corroded surfaces and corrosion products were sealed and fixed by epoxy resin, and the cross-section of the iron was acquired by milling to the desired part using the coarse sandpaper. The element contents of corroded surfaces were semi-quantified with a build-in energy dispersive spectrometer (EDS) of the SEM equipment.

### Electrochemical studies of the samples after immersion in different media

All the electrochemical experiments were performed with an electrochemistry station CHI760E (Chenhua, Shanghai). A three-electrode system was used with the saturated calomel electrode as a reference electrode and the Pt electrode as a counter electrode. Before the potentiodynamic scanning tests, the steady open circuit potential (OCP) was acquired and the scanning potential range was set according to OCP. The potential was swept from the value of OCP minus 400 mv to the value of OCP plus 400 mv and the scanning rate of potential was 0.33 mv s^−1^. For the specimen immersed in HS, the potential was swept to the value of OCP plus 500 mv to evaluate the passivation behavior. The work electrodes had an area of 1 cm^2^. Tafel curves of iron after immersed for 3 days in different media were acquired in the electrochemical workstation. Based on the Tafel curve, the corrosion potential and the corrosion current were calculated by the software equipped in the workstation. Electrochemical impedance spectra (EIS) were also measured in different media at 37°C. The potential was set as the OCP and the frequency was scanned between 0.01 and 100 000 Hz. The perturbation amplitude of the frequency was 5 mV. The software ZSimpWin was used to analyze the data to acquired corrosion parameters of iron.

### Measurements of corrosion mass (*M*_corro_) of iron in five media

An inductive coupled plasma emission spectrometer (iCAP 7400, America) was used to acquire the corrosion mass (*M*_corro_) of iron samples after immersion in the five aqueous media. Prior to the measurements, the corrosion products were removed and dissolved by hydrogen chloride solution with 3.5 g hexamethylenetetramine per liter. The solutions were collected for the measurement. Standard ferrous solutions with different concentrations were also be prepared and measured for the establishment of the standard curve to calculate the corrosion products mass of the iron immersed in different media.

### Statistical analysis of the corroded surfaces

Three independent samples in each group were examined in the ICP tests and potentiodynamic scanning tests. In the results of corrosion surface for corrosion coverage, more than three images were used for the calculation and acquired the corrosion coverage (θ) of each specimen. The pit depth was also measured based on the images of cross-section. More than 10 pits were measured for each cross-sections in DI, NS and AS to acquire the average pit depth of iron after immersion for 3 and 7 days. More than 5 pits were measured in HS and PBS considering the small pit amount on the whole surface. For the statistics of corrosion pit radius and amount, more than 3 images were used in every sample. The number of specimens for each group will be indicated in each of the corresponding figures. The results of corrosion coverage, corrosion mass, corrosion current, corrosion radius and corrosion pit depth were all expressed as mean ± standard deviation (SD).

## Results

### Derivation of the C-M equation and definition of the half-coverage mass *M*_corro50%_ as the new inhomogeneity parameter

While corrosion rate can be simply obtained by the first order derivative of corrosion mass over corrosion time, there is so far no unified parameter to quantify corrosion inhomogeneity. To quantify corrosion inhomogeneity, we attempt to firstly establish a function between corrosion coverage θ and corrosion mass per initial surface *M*_corro_, namely,

(1) θ=fMcorro

**Figure 2.**
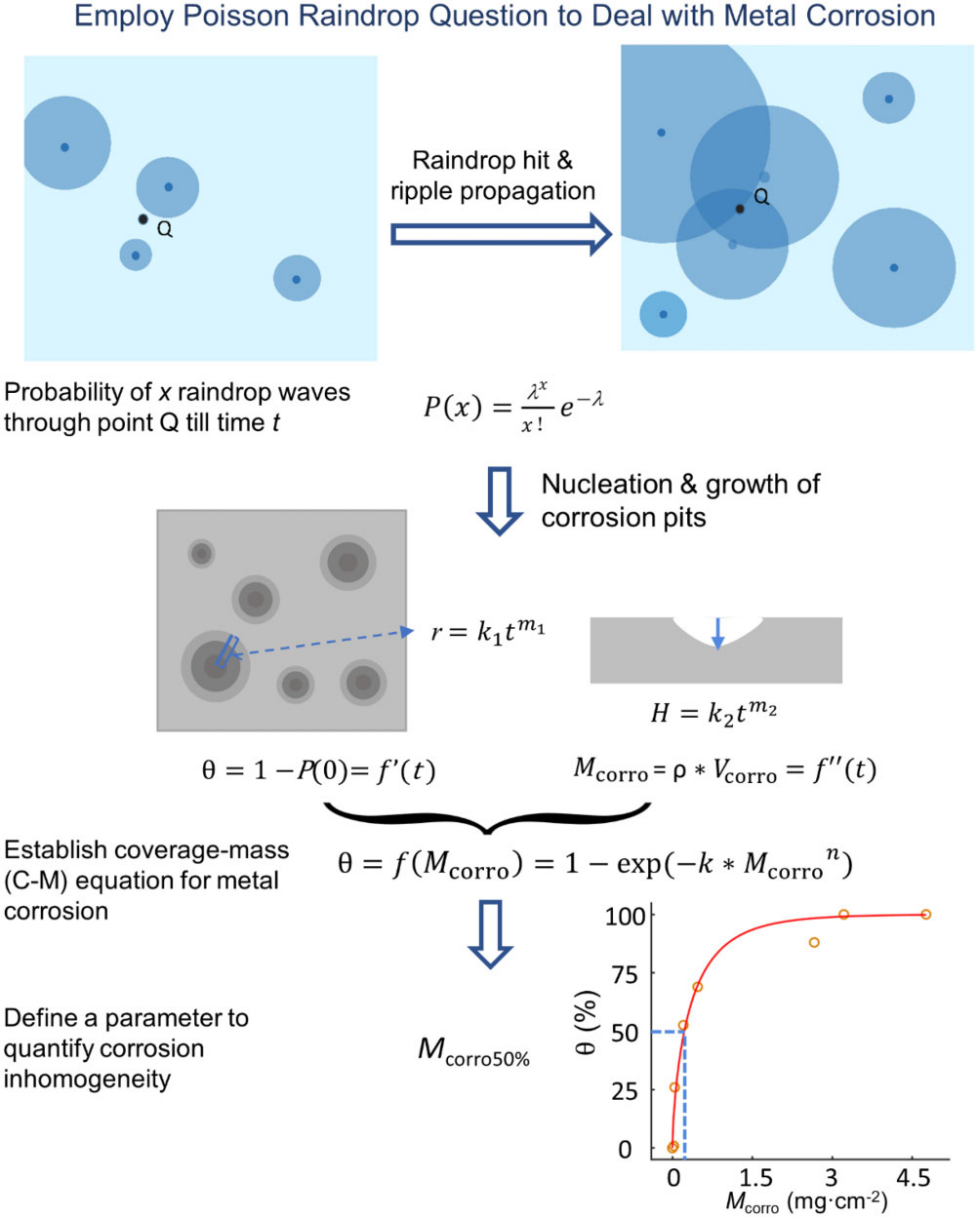
Introduction of Poisson raindrop question to analyze the corrosion process and the derivation of C-M equation to quantify the corrosion inhomogeneity. The corrosion coverage θ is defined as the projected area of all of the corroded units, say, corrosion pits, over the whole projected area.

The nucleation of corrosion pits is a stochastic process in some aspects [16, 17]. We anticipated that the spatiotemporal distribution of the corrosion pit or the corrosion unit on the corroded surface be described by a ‘Poisson raindrop question’, as schematically presented in Fig. 2.

We set *x* as the number of ‘ripples’ or ‘raindrop waves’ passing through a site Q at time *t*. Then its probability *P* obeys Poisson equation:

(2) Px=(λx/x!)e-λ 

Here, *λ* is the average number of ‘raindrop waves’ or corrosion pits passing through the random point Q.

The probability at which no corrosion pit passes through point Q or the point Q remains uncorroded is

(3) P0= e-λ 

Thus, the probability of any corrosion pit passing through point Q or the corrosion coverage (θ) of the corroded surface could be expressed as

(4) θ=1 – e-λ

Then, the calculation of corrosion coverage (θ) is committed to that of *λ*, the average number of corrosion pits passing through Q. Some assumptions and simplifications are made for the derivation, and the detailed derivation of *λ* is shown in the Supplementary data.

In brief, the corrosion process contains nucleation and growth. Corrosion pits are assumed to nucleate in a homogeneous way. So, the pit number *N*(*t*) = *ct*, where *t* is the corrosion time and *c* is the average speed of pit nucleation per initial surface. The kinetics of pit growth is assumed as $k_{1}t^{m_{1}}$ in radius and $k_{2}t^{m_{2}}$ in depth. The *k*_1_ and *k*_2_ are the growth constants along the dimensions of radius and depth, respectively, while *m*_1_ and *m*_2_ indicate the corresponding pit growth exponents.

As a result, the time-dependent corrosion coverage is, under homogeneous nucleation, written as

(5) θt=1-e-π2m1+1ck12t2m1+1

It can be simplified as

(6) θ=1-exp⁡-k'tn'

We further calculated corrosion volume (*V*_corro_) with its detail shown in the Supplementary data. The corrosion mass per initial surface (simply called corrosion mass) is then expressed as

(7) Mcorro=ρVcorro=πρck12k2t2m1+m2+12m1+m2+1

Here, *ρ* is the density of metal. This equation can be simplified as

(8) Mcorro=atb

The values of θ and *M*_corro_ of iron after different immersion time in DI are shown in Supplementary Fig. S2, and the experimental data are well fitted by (6) and (8), which confirms our theoretical derivation.

Combination of (5) and (7) leads to elimination of the corrosion time *t*. Eventually, we obtain the relation between the corrosion coverage (θ) and corrosion mass (*M*_corro_) as

(9) θ=1-exp⁡-k×Mcorron

Such an equation is named by us as the coverage-mass equation and abbreviated as C-M equation. In this equation, *k* is a complicated parameter pertinent to the parameters of *k*_1_, *k*_2_, *m*_1_, *m*_2_ and *c*; and *n* is a scaling exponent related to the kinetics of nucleation and growth of corrosion pits.

Based on the C-M equation, we put forward a parameter *M*_corro50%_ defined as the corrosion mass (*M*_corro_) when the corrosion coverage (θ) reaches 50%. This inhomogeneity parameter, half-coverage mass, can be calculated by the fitted *n* and *k* with the equation

(10) Mcorro50%=ln2k1n

### Confirmation of the C-M equation and the half-coverage mass *M*_corro50%_ by the corrosion behavior of iron in DI

The data of corrosion coverage (θ) and corrosion mass (*M*_corro_) of iron in DI well obey (9), namely, the C-M equation, as shown in the right lower of Fig. 2. The *M*_corro50%_ per initial surface resulted in 0.22 mg cm^−2^.

**Figure 3.**
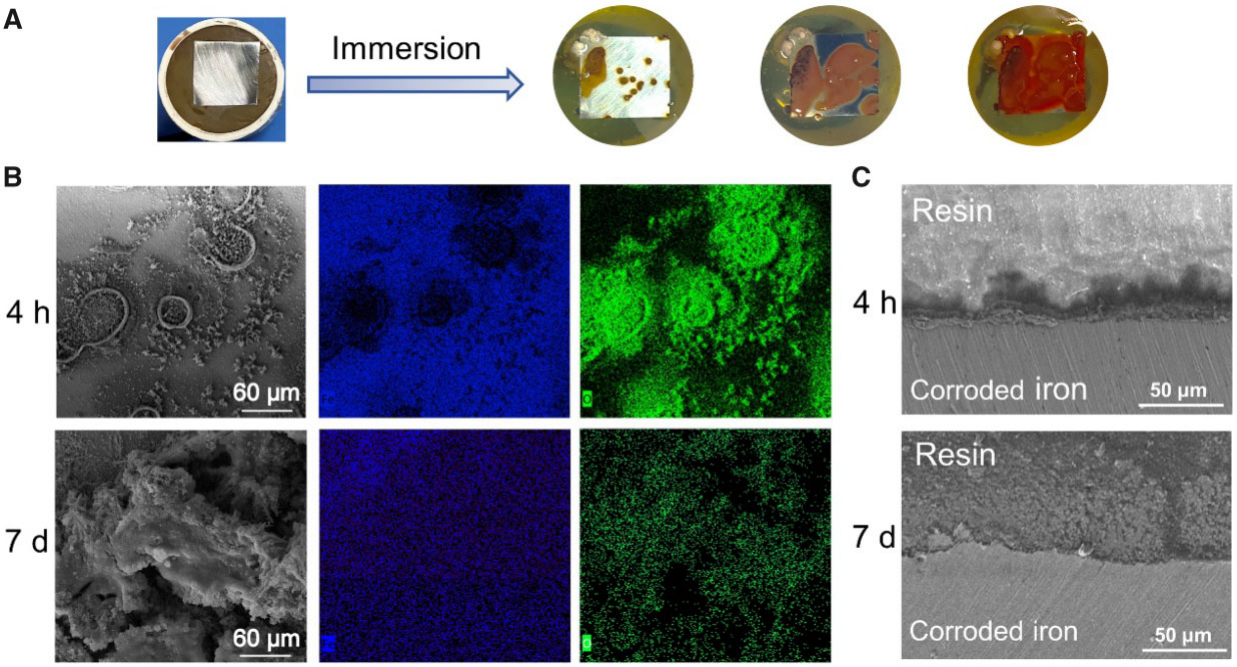
Corrosion behavior of iron after immersion in deionized water (DI). (**A**) Global view of an iron sheet before and after immersion in DI with time increased. (**B**) SEM images of iron surface after immersion for 4 h and 7 days in DI and the corresponding EDS mapping of the corroded iron. (**C**) The SEM images of cross-sections of iron after immersed in DI for 4 h and 7 days. A resin was used to seal the corroded surface, and the sealed iron sheet was milled to enable observation of a cross-section.

Figure 3 demonstrates the corroded iron sheets after immersion in DI at different time. The global view is shown in Fig. 3A, indicating the increase of coverage of corrosion products with time. The EDS mapping images of iron after immersion for 4 h and 7 days in DI are presented in Fig. 3B. As immersion time increased, the corrosion coverage increased and oxide was generated on the surface of iron. As shown in Fig. 3C, corroded iron at 7 days exhibited significantly larger and deeper corrosion pits compared to that of 4 h.

We also measured EIS and potentiodynamic polarization curves. A typical Tafel curve of iron after immersion for 3 days is shown in Supplementary Fig. S3. The resultant average corrosion current read 10.1 μA cm^−2^ and the corrosion potential was −0.57 V. The time-dependent charge transfer resistance *R*_ct_, which indicates corrosion resistance of the sample, was acquired from EIS and also presented in Supplementary Fig. S3. *R*_ct_ decreased firstly and increased with time after 1 day because the corrosion products hindered the later mass transfer.

### Iron corrosion behaviors in other four media

Besides DI, some biomimetic media containing ions such as NS, PBS and HS are more useful, for iron is a promising biomaterial [18–20]. Iron corrosion in AS was also examined as an extensive study, because AS is an important medium in metal applications in the fields of construction and transportation, etc. [21]. The application fields and the ion composition of these media are listed in Table 1 and Supplementary Table S1. These media were chosen in our investigation of iron corrosion to reflect different environments of metal applications, especially for the biomaterials.

**Table 1.** Description of the five aqueous media examined in this study

	Composition and character	Fields of application
DI (deionized water)	Purified water free of ions	Chemistry, physics and biology
AS (artificial seawater)	Solution with ion types and concentrations similar to those of seawater	Industry, agriculture, transportation and military
NS (normal saline)	A sodium chloride solution with the same osmolarity as that of human body fluid	Biology and medicine
PBS (phosphate-buffered saline)	A phosphorus saline buffer solution with the same pH and osmolarity as those in human tissue fluid	Biomaterials, pharmacy and regenerative medicine
HS (Hank’s solution)	A saline buffer solution with the same pH, ion types and concentrations as those in mammal plasma	Medical stents and scaffolds in contact with blood

**Figure 4.**
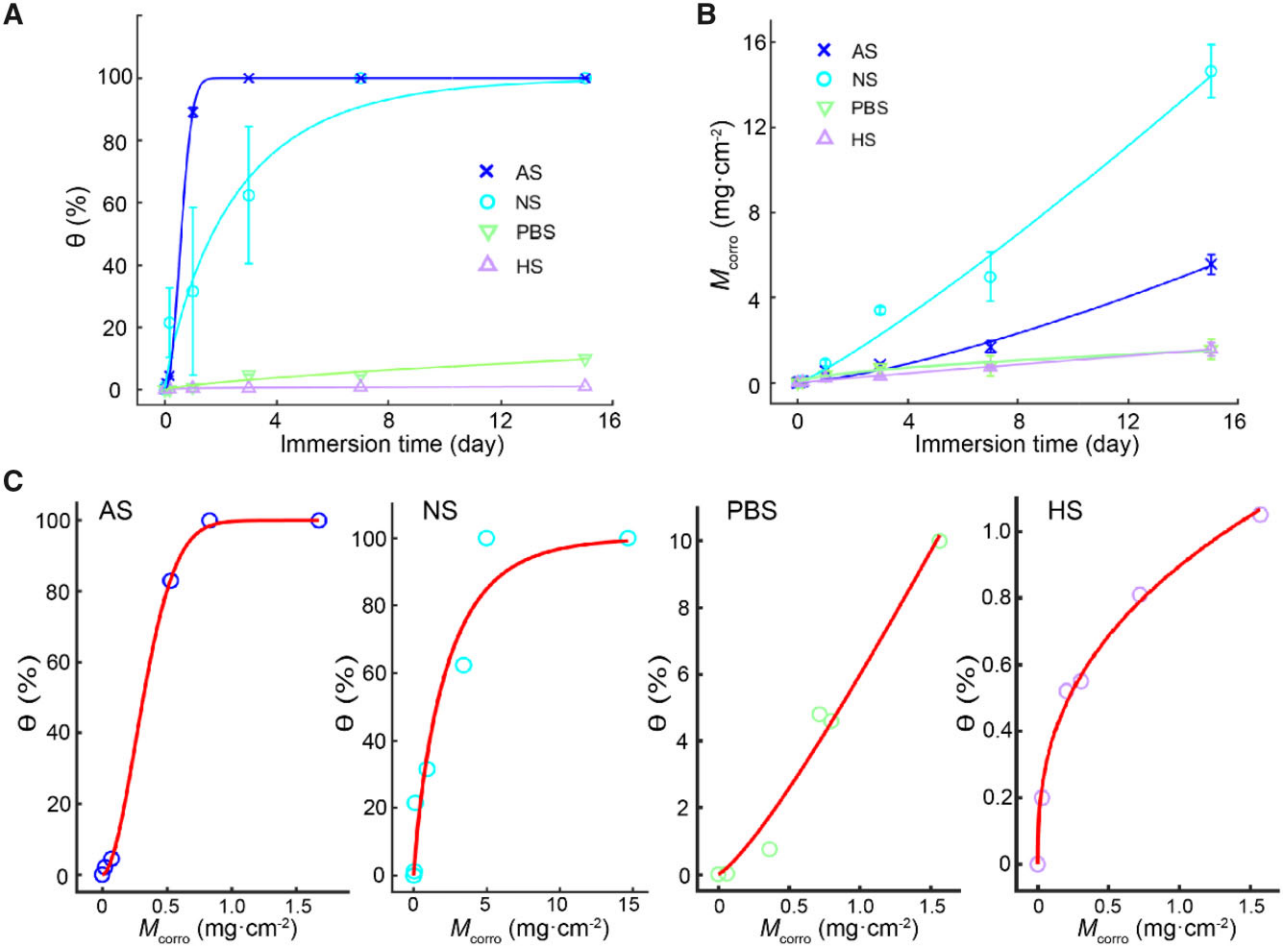
Fitting of corrosion data of other four media. (**A**) Corrosion coverage (θ) and the fitted lines of data with (6) in the indicated four media. Error bars represent SD from independent experiments in each group, and *n *=* *3 for each group. The fitting parameters of *k*′ and *n*′ are shown in Supplementary Table S2. (**B**) Corrosion mass (*M*_corro_) of iron and fitted lines of data with (8) in the indicated four media. The fitting parameters *a* and *b* are shown in Supplementary Table S3. (**C**) Fitting of *M*_corro_ versus θ by (9), namely, C-M equation. The fitted parameters of *k* and *n* are listed in Supplementary Table S4.

Resultant data of θ and *M*_corro_ and the fitted curves in the four ion-containing media are shown in Fig. 4A and B. Among the five media (DI, AS, NS, PBS and HS), iron immersed in NS had the largest corrosion rate but not the fastest coverage of corrosion area. In AS, the iron had the fastest corrosion coverage rate but not the fastest mass rate. In PBS and HS, the iron exhibited both smaller corrosion coverage and corrosion rates. The corrosion coverage (θ) versus corrosion mass (*M*_corro_) of iron corrosion in each of the different media obeys the C-M equation, as shown in Fig. 4C.

Global views of iron corrosion in different media are shown in Supplementary Fig. S4. As a demonstration, Supplementary Fig. S5 presents SEM images before and after removal of corrosion products of iron in PBS for 4 h and 7 days, which clearly illustrate the additional generation of corrosion pits with time and thus a homogeneous nucleation on the iron surface.

**Figure 5.**
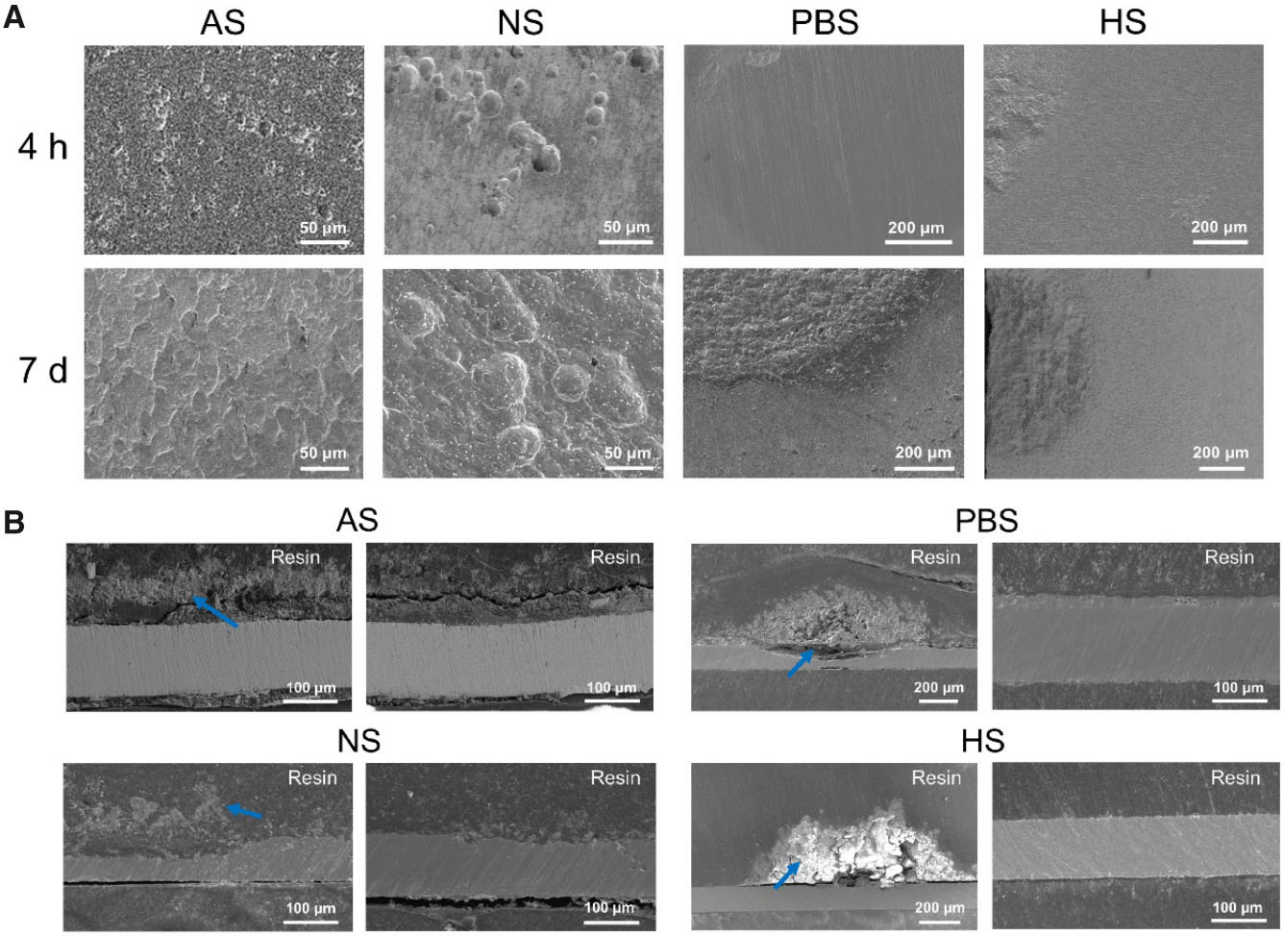
Corrosion morphology of iron in the other four media. (**A**) SEM images of iron surfaces after removal of corrosion products. (**B**) SEM images of cross-sections of iron after immersion for 7 days. The blue arrows point to some typical corrosion products of iron. Two images are shown for each medium, indicative of corrosion inhomogeneity.

The coverage θ could be calculated based on either the optical images via global observation with corrosion products or multiple SEM images after removal of corrosion products. Some typical SEM images of corroded surfaces after removal of corrosion products are shown in Fig. 5A. More or less corrosion pits were observed on iron surfaces after 4 h of immersion in all of the media. After immersion for 7 days, corrosion products almost covered the whole area of the iron in AS and NS. In PBS and HS, the corrosion pits remained in a small amount and grew larger.

We also observed the cross-sections of the iron during corrosion. In Fig. 5B, the pits were hardly seen in AS, while the corrosion pits were observed clearly in NS. The pits on iron sheets immersed in PBS and HS were much deeper; nevertheless, there were also some parts with low corrosion extents observed in the cross-section, indicative of significant corrosion inhomogeneity.

We employed EDS techniques to examine the element distribution of the corroded surfaces, and typical SEM-EDS images in the four media are presented in Supplementary Figs. S6–S9. Specifically, we examined the background elements with the region of interest indicated by the blue boxes in the SEM images in Supplementary Fig. S10, and the resultant chemical compositions are listed in Supplementary Table S5. On the iron surfaces after immersed in AS for 4 h, magnesium and carbonate ions deposited on the uncorroded surfaces. After immersion for 7 days, the corrosion surface of iron contained element Mg, indicating that magnesium ions took part into the corrosion products. NS immersion resulted in the corrosion products similar to DI immersion. In the cases of iron corrosion in PBS and HS, the phosphorus element was detected, indicating phosphate deposition on the surfaces. In HS, elements Ca and C were also detected on iron surfaces.

What is more, potentiodynamic tests were conducted after immersion for 3 days, and the Tafel curves, the corrosion potentials and corrosion currents are shown in Supplementary Fig. S11. The corrosion rates measured by the electrochemical method were basically consistent with that by measuring time dependence of corrosion mass.

In EIS experiments, charge transfer resistance *R*_ct_ was acquired, and the results are shown in Supplementary Fig. S12. In PBS and HS, *R*_ct_ increased with time and much larger than those in NS, AS and DI, indicating the formation of passivation films after immersion in the former two media and thus hindering the corrosion process.

### Comparison between pitting factor and half-coverage mass to quantify corrosion inhomogeneity

**Figure 6.**
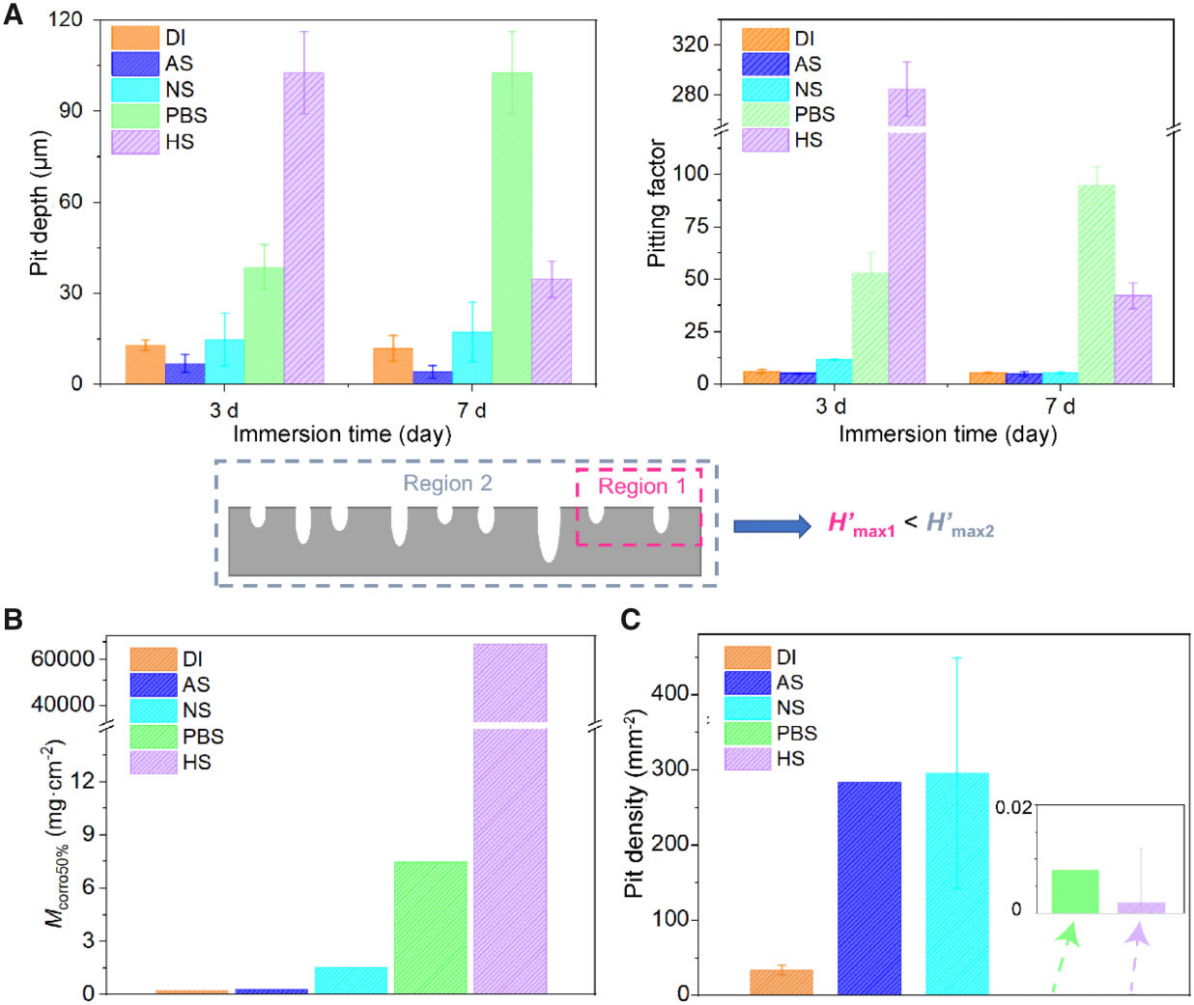
Comparison of the inhomogeneity-relevant parameters among iron corrosion in five media. (**A**) Corrosion pit depths and pitting factors of iron immersed for 3 and 7 days in different media. The lower picture shows schematically the fluctuation of corrosion pit depths with the examined regions. (**B**) Half-coverage mass *M*_corro50%_ calculated using C-M equation to quantify the corrosion inhomogeneity of iron in the indicated 5 media shown in sequence of increased corrosion inhomogeneity. (**C**) Corrosion pit density after immersion for 4 h to characterize corrosion inhomogeneity.

We also examined the pitting factor of iron corrosion and made comparison with our half-coverage mass. Pitting factor is defined as the depth of the deepest corrosion pit divided by the average corrosion depth calculated from corrosion rate or corrosion mass [16]. The measured pitting depths in our experiments and the calculated pitting factors are shown in Fig. 6A. The new corrosion inhomogeneity parameter *M*_corro50%_ calculated based on fitting with C-M equation is shown in Fig. 6B, and the media in the horizontal coordinate follow the sequence of the increased inhomogeneity.

Besides dependence of the so-called deepest pit upon the region scale as indicated in the lower presentation in Fig. 6A, the measured pitting factor in HS on day 7 is even smaller than that on day 3, which came from the different cross-sections and reflected the nonconvergent or unstable character of this transitional parameter. In contrast, our corrosion inhomogeneity parameter *M*_corro50%_ is mathematically more convergent or stable and less cost-effective in experiment. We also show corrosion pit density after immersion for 4 h in Fig. 6C. Combination of half-coverage mass and pit density can afford a more comprehensive physical picture of corrosion inhomogeneity.

## Disscusion

Temporal and spatial evolution of matter is universal in materials science and engineering, and the kinetic study is of vital importance [22, 23]. Corrosion inhomogeneity influences material properties significantly [24, 25] and thus affects the application of the material. Metals are wildly used material [26–30] and most of them faced with the problem of corrosion. Besides the study of approaches of metal protection [31], the corrosion behavior [32, 33] is also a fundamental research topic. In the present study, we propose the C-M equation and the half-coverage mass for quantification of corrosion inhomogeneity and discuss the corrosion behavior on corrosion inhomogeneity as well as corrosion rate.

### C-M equation and the corrosion inhomogeneity parameter *M*_corro50%_

After the deduction of the C-M equation, we find that the equation has a form similar to the Avrami equation. The Avrami equation has been used in the crystallization of metal, ceramic and polymer [34–36]. It has the general form of

(11) φ=1-e-ktn

Here, *φ* is the volume fraction of crystallization, *k* is the speed constant, which is related to the nucleation and growth speed of the crystallization process and *n* is the Avrami exponent, which is relevant to the phase transformation mechanism.

In our C-M equation, *k* is a comprehensive parameter to characterize the corrosion inhomogeneity in some aspect, and *n* is a parameter related to the radius and depth growth exponents.

In the case of homogeneous nucleation, *k* is expressed as

(12) $$\mathrm{lg}k=\frac{m_{2}}{2m_{1}+m_{2}+1}\;\mathrm{lg}(\pi c{k_{1}}^{2})+\frac{{2m}_{1}+1}{{2m}_{1}+m_{2}+1}lg\frac{2m_{1}+m_{2}+1}{k_{2}\rho }\;-\mathrm{lg}{(2m}_{1}+1)$$

And the exponent of the C-M equation is expressed as

(13) n=2m1+12m1+m2+1

Otherwise, in the case of heterogeneous nucleation, the pit density is a constant independent from immersion time and thus denoted as *N*_0_. Then, the time dependence of corrosion coverage (θ) and corrosion mass per area (*M*_corro_) can be expressed as

(14) θt=1-e-N0k12t2m1

and

(15) Mcorro=πρN0k12k2t2m1+m2

The detailed formula derivation is shown in the Supplementary data.

In this case, we also eliminated *t* and gained the relation between θ and *M*_corro_ by (9), and *k* and *n* are, respectively, written as

(16) lgk=m22m1+m2lg⁡(N0k12)-2m12m1+m2lg⁡(πρk2)

and

(17) n=2m12m1+m2

In our deduction of *k* and *n*, the value of *n* should be smaller than 1 in any case. But by fitting of the experimental data of iron corrosion in the five different media, the values of *n* were larger than 1 in AS and PBS, as shown in Supplementary Table S4. In our assumption, a corrosion pit always grows with time once generated. But in the immersion experiment, the resistance increased with time later due to the passivation by corrosion products sometimes, leading to slow down corrosion at the late stage. Some corrosion pits might even be repassivated along with ion deposition and product formation, which interpret the derivation of the fitted parameters to some extents.

After showing the relation between the nucleation and growth parameters and the fitted *k* and *n*, the effect of corrosion kinetic parameters on the *M*_corro50%_ in different media could be analyzed. The values of *M*_corro50%_ in DI, AS and NS were relatively smaller mainly because of a larger *c* (the average speed of pit nucleation per initial surface), as shown in the pit density in Fig. 6C. And in NS, *M*_corro50%_ was larger than those of AS and DI for the larger *k*_2_, which indicated a faster pit depth growth as well as a deeper pit. In HS and PBS, *M*_corro50%_ were much larger mainly because of the smaller *c* and larger *k*_2_ as the larger pit depth and smaller pit density in Fig. 6A and C. And in HS, as the smaller value of *n*, the larger *m*_2_ might also contribute to the larger *M*_corro50%_.

As shown in (6), the relation between corrosion coverage θ and corrosion time *t* is also similar to the Avrami equation. Here, in the case of homogeneous nucleation

(18) n′=2m1+1

(19) k′=π2m1+1ck12

And in the case of heterogeneous nucleation

(20) n′=2m1

(21)  k′=N0k12

In the case of homogeneous nucleation, the parameters *a* and *b* in (8) can be expressed as

(22) a=πρck12k22m1+m2+1

(23) b=2m1+m2+1

and in the case of heterogeneous nucleation as

(24) a=πρN0k12k2

(25) b=2m1+m2

The actual corrosion might be initiated via a mixing of complete homogeneous and heterogeneous nucleation. Nevertheless, the nucleation mechanism influences only the coefficents of (9). The key equation (C-M euqation) and the inhomogeneity parameter (*M*_corro50%_) work in all of cases.

We ever tried to measure *t*_50%_, the time when corrosion coverage reaches 50%. The results in different media are shown in Supplementary Fig. S13. Although basically consistent with *M*_corro50%_, *t*_50%_ neglects the pit growth in depth and cannot reflect the corrosion inhomogeneity comprehensively. So, it is *M*_corro50%_ that captures the essence of the corrosion heterogeneity.

As the new corrosion inhomogeneity parameter is concerned, the value of *M*_corro50%_ in HS was 10 000-fold larger than even the second serious case in the five media (Fig. 6B). In this case, the coverage data joining in fitting are small (Fig. 4C). The ability by using small coverages to calculate *M*_corro50%_ made the application of the C-M equation to determine corrosion inhomogeneity parameter *M*_corro50%_ in a more convenient and universal way.

Our suggested parameter half-coverage mass *M*_corro50%_ is better than the conventional pitting factor owing to the following reasons: (i) more comprehensive description of the extent of corrosion inhomogeneity; (ii) independent upon observation area in principle and thus stable and reliable in experimental measurements; and (iii) free of cross-section SEM and thus facile and cost-effective.

### Independence of corrosion rate and corrosion inhomogeneity

**Figure 7.**
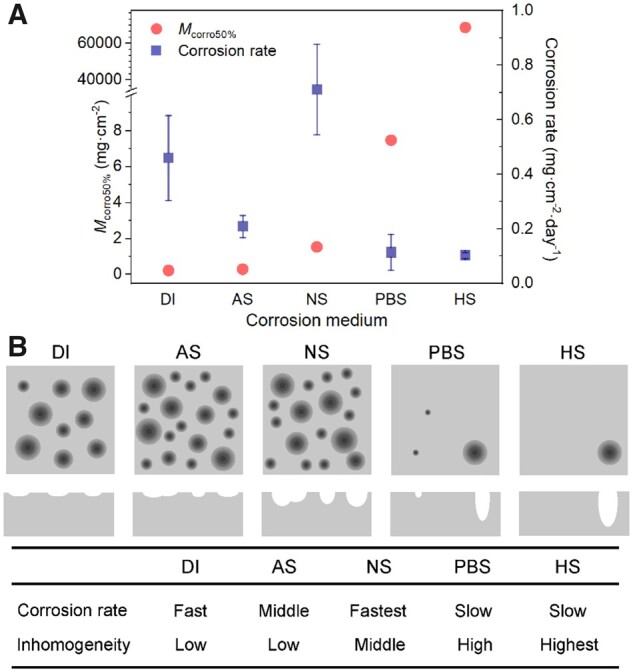
Summary of inhomogeneity parameters and corrosion rates of iron in different media. (**A**) Experimental data of corrosion rates and calculated *M*_corro50%_ in the indicated five media. (**B**) Comparison of corrosion rates and inhomogeneity extents of iron in the different media. Both the top views and cross-sections are schematically presented. It is worthy of noting that the corrosion rate is strongly dependent of corrosion time, and the description in the lower part is based on 7 days of observation.

We calculated corrosion rates from the time dependence of corrosion mass (*M*_corro_). The results are shown in Supplementary Fig. S14. The corrosion rate and corrosion inhomogeneity (*M*_corro50%_) are shown in the same graph of Fig. 7A, and no definite relevance was observed between them. It illustrates that the corrosion inhomogeneity and corrosion rate are two parameters to quantify the corrosion behaviors independently.

It is surprising that the largest corrosion rate is about 7-fold of the smallest one and the highest *M*_corro50%_ is about 300 000-fold of the smallest one in the five media examined in this study. What is more, the most serious inhomogeneity does not correspond to the fastest corrosion. So, the corrosion inhomogeneity must be seen as an irreplaceable aspect and should be more emphasized in evaluation of corrosion behaviors. After analysis of iron corrosion in the five different media, the different corrosion behaviors are schematically summarized in Fig. 7B.

### Effects of medium composition on corrosion rate and inhomogeneity

**Figure 8.**
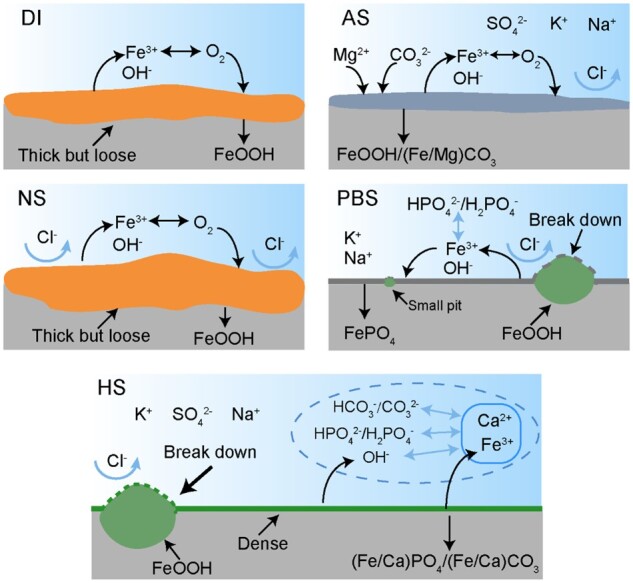
Schematic presentation of iron corrosion in the indicated five typical aqueous media.

Now we discuss the effects of ions in different media on the corrosion of iron, as schematically presented in Fig. 8. In DI, iron corroded via the simple anodic reaction and cathodic reaction of iron loss electrons and oxygen gain electrons in 7 days. In NS, the chloride ion could take part in iron corrosion and make the corrosion products looser and less protective [37, 38]. Chloride ions accelerated corrosion globally, especially pit growth in depth. In AS, magnesium and carbonate ions deposited on the surface, forming a non-protective film on iron and leading to the passivation of corrosion pits, while the high concentration of chloride ions in AS may result in fast nucleation of corrosion pits. So, the corrosion process in AS was less heterogeneous than in NS.

In PBS, ferric ions dissolved by iron corrosion and phosphate ions in the virgin solution could form a passive film and decrease the corrosion rate [39–41]. Nevertheless, the passive film was likely to break down locally in the presence of chloride ions. Because of the larger cathodic area and the acidification in the pit, the growth along the dimension of pit depth as well as radius increased further. In the late stage of corrosion, the passive film probably cracked and other corrosion pits were generated. In HS, a passive film was formed with depositing of carbonate, calcium ion and phosphate ion, which decreased the corrosion rate of iron [42]. In the presence of calcium ion, the deposited passive film would be more protective than in PBS [43, 44]. Yet, chloride ions might act to break the passive film and thus form local corrosion pits, leading to rapid growth along the dimension of pit depth. Due to the denser passive film than PBS, the corrosion coverage in HS was smaller in the late stage, and the corrosion inhomogeneity in HS was much significant. These could interpret the difference between the corrosion inhomogeneity of iron in PBS mimicking tissue fluid and in HS mimicking blood. Since NS, PBS and HS are popular media in biology and medicine, the present study is particularly helpful for the research and development of biomedical materials.

### Limitations

To establish the analytic expression between corrosion coverage (θ) and corrosion mass (*M*_corro_), the nucleation of the corrosion pit and its growth kinetic were assumed. But the repassivation of the corrosion pit during corrosion was ignored in the theoretical deduction. The deposition of corrosion products might change the growth kinetic of corrosion pits and thus lead to the deviation between theory and experiment. Nevertheless, the basic parameter *M*_corro50%_ still work irrespective of the concrete analytical forms of the exponents of C-M equation, namely, *k* and *n*.

It is worthy of emphasizing again that the C-M equation enables the fitting of *M*_corro50%_ using a few experimental data with coverage even much less than 50%, which affords, in principle, a unified criterion to compare the extents of corrosion inhomogeneity of metals in different media.

Besides pitting corrosion, there are other forms of local corrosions of metals, such as crevice corrosion, galvanic corrosion and intergranular corrosion, for different metals under different mechanisms. The kinetics of a local corrosion might not exactly follow the C-M equation if the corrosion unit is insufficiently small or stochastic. Nevertheless, the inhomogeneity parameter *M*_corro50%_ still works if the statistical view field is significantly larger than the local corrosion units.

### Potential extendibility of the present fundamental investigation

Besides iron, there are many other metals widely used and faced with corrosion [45–47]. The C-M equation and the new inhomogeneity parameter can also be used to analyze the anti-corrosion performance, guide metal selection, assist corrosion management, estimate the economy loss and prevent the disaster caused by corrosion. With the development of biomaterials [48–53], the biodegradable type has been the front of medical devices [54–57], and some corrodible metals have been tried as the skeletal materials [58–60]. Even a metal-polymer composite stent has been into clinical research [61, 62]. Our equation is expected to be a new tool to investigate biodegradable medical devices and assist the evaluation and choice of the biomaterial. Some other biodegradable materials such as polymer, glass and ceramics [63–67] could also be evaluated by our equation for characterization of degradation inhomogeneity.

What is more, we expect that the equation may be extended into other fields beyond material sciences such as the diversity of microorganisms in waste water [68], gene mutation [69], the evolution of mantle [70], prevention and control of endemic diseases [71]. Our basic research calls on the attention on the time-dependent spatial heterogeneity for many other complex processes even beyond natural science and engineering, for instance, the cluster evolution in dynamics of finance and economics, population and social organization.

## Conclusions

The C-M equation was proposed and derived by us to describe the corrosion behavior. Half-coverage mass *M*_corro50%_ was defined to quantify the corrosion inhomogeneity of metal corrosion. The theoretical equation was confirmed by our experiments of iron in five aqueous media. The new parameter was justified in the characterization of corrosion inhomogeneity. We have also demonstrated that the corrosion inhomogeneity parameter *M*_corro50%_ cannot be reflected by corrosion rate. While the largest corrosion rate is about 7-fold of the smallest one, the highest *M*_corro50%_ is about 300 000-fold of the smallest one, and the most serious heterogeneity does not correspond to the fastest corrosion. Inhomogeneous corrosion frequently occurs in biodegradation of biomaterials, and the quantification of extents of inhomogeneity should be more emphasized and the equation will be useful and meaningful in evaluation and development of biomaterials.

## Supplementary Material

rbad007_Supplementary_Data
