# Supplementary material for: Establishment of coverage-mass equation to quantify the corrosion inhomogeneity and examination of medium effects on iron corrosion
Source: Regen Biomater. 2023 Feb 1;10:rbad007. doi: 10.1093/rb/rbad007 (PMC9933843; doi:10.1093/rb/rbad007)
Supplement: rbad007_Supplementary_Data [file rbad007_supplementary_data.pdf]

# Supporting Information

## Establishment of coverage-mass equation to quantify corrosion inhomogeneity and examination of medium effects on iron corrosion

*Xin LI, Jiandong DING**

State Key Laboratory of Molecular Engineering of Polymers, Department of Macromolecular Science, Fudan University, Shanghai 200438, China

* Corresponding author. E-mail address: jdding1@fudan.edu.cn (J.D. Ding)

This PDF file includes Supplementary Formula Derivation, Figures S1 to S14, Tables S1 to S5. The supplementary figures and tables are shown in sequence mentioned in the main manuscript.

## Supplementary Formula Derivation

The corrosion process with time $t$ is divided into two stages, nucleation and growth. The pit nucleation is presumed to be generated in a homogeneous way with the kinetic $ct$ or in a heterogeneous way of a constant $N_0$. In both cases, the pit radii are assumed to grow with $k_1 t^{m_1}$ and the average pit depths are assumed to grow with $k_2 t^{m_2}$.

The corrosion nucleation is regarded by us as a stochastic process and described by the Poisson raindrop question. As schematically presented in Figure 2 in the main manuscript, if the number of raindrop (or corrosion pit) passing through a random point Q is denoted as $x$, its probability $P(x)$ must follow the Poisson distribution with the form of equation (2) in the main manuscript. The uncorroded area is thought to be the region with zero "raindrop wave". The corrosion coverage (θ) is thus expressed as equation (4), and could be acquired by calculation of $\lambda$, the average number of "raindrop waves" or corrosion pits passing through point Q.

We derive the equation first for the case of homogeneous nucleation. In a "ripple" ring with the distance $L$ from point Q, only the corrosion pits whose generation time is larger than $(\frac{L}{k_1})^{\frac{1}{m_1}}$ could pass through Q. So

$$\mathrm{d}\lambda = c\left[t - \left(\frac{L}{k_1}\right)^{\frac{1}{m_1}}\right] 2\pi L dL \tag{S1}$$

After integration, $\lambda$ is expressed as

$$\lambda = \int_0^{k_1 t^{m_1}} c\left[t - \left(\frac{L}{k_1}\right)^{\frac{1}{m_1}}\right] 2\pi L dL = \frac{\pi}{2m_1 + 1} c {k_1}^2 t^{2m_1+1} \tag{S2}$$

Combination of this equation with equation (4) leads to equation (5) describing corrosion coverage (θ) *versus* corrosion time $t$.

The relation between θ and $t$ is thus similar to the Avrami equation with the form of equation (6).

Then we deal with corrosion mass per initial surface ($M_{\mathrm{corro}}$). In the case of homogeneous nucleation, the variation of pit density $N(t)$ could be written as $\Delta N(t) = c\Delta t$.

The time $t$ is related to $r$ as

$$r = k_1 t^{m_1} \tag{S3}$$

So $\Delta t$ between $r \sim r + \Delta r$ is expressed as

$$\Delta t = \Delta \left(\frac{r}{k_1}\right)^{\frac{1}{m_1}} \tag{S4}$$

And the amount of the corrosion pits between $r$ and $r+\Delta r$ is written as

$$\Delta N(r) = c\Delta \left(\frac{r}{k_1}\right)^{\frac{1}{m_1}} \tag{S5}$$

The variation of the corrosion volume $\Delta V_{\mathrm{corro}}$ is expressed as

$$\Delta V_{\mathrm{corro}} = \pi r^2 k_2 \left(\frac{r}{k_1}\right)^{\frac{m_2}{m_1}} c\Delta \left(\frac{r}{k_1}\right)^{\frac{1}{m_1}} \tag{S6}$$

And the corrosion volume per unit area of iron could be expressed as

$$V_{\mathrm{corro}} = \int_0^r \pi r^2 k_2 \left(\frac{r}{k_1}\right)^{\frac{m_2}{m_1}} c\mathrm{d}\left(\frac{r}{k_1}\right)^{\frac{1}{m_1}} = \frac{\pi c {k_1}^2 k_2 t^{2m_1+m_2+1}}{2m_1 + m_2 + 1} \tag{S7}$$

The corrosion mass ($M_{\mathrm{corro}}$) per area of iron is thus obtained as shown in equation (7).

We eliminate the time $t$ after combination of equations (5) and (7) and thus obtain equation (9), namely, the C-M equation.

Now we discuss the case of heterogeneous nucleation, which occurs, for instance, around some preformed defects. In this case, the nucleation number is a constant, denoted as $N_0$. Then, the average number of raindrop waves or corrosion pits passing

through a random point Q at time $t$ is integrated as

$$\lambda = \int_0^{k_1 t^{m_1}} N_0 2\pi L dL = \pi N_0 {k_1}^2 t^{2m_1} \tag{S8}$$

After combining equation (4), corrosion coverage (θ) as a function of time is written as equation (14).

As for the corrosion volume and thus mass in the case of heterogeneous nucleation, all the corrosion pits are generated at the same time. So the corrosion volume per area of iron could be calculated by corrosion pit density multiply the volume of the single corrosion pits

$$V_{\mathrm{corro}} = N_0 \pi {k_1}^2 t^{2m_1} k_2 t^{m_2} = \pi N_0 {k_1}^2 k_2 t^{2m_1+m_2} \tag{S9}$$

The corrosion mass ($M_{\mathrm{corro}}$) of the unit $cm^2$ area of the metal is thus written as equation (15).

Combination of equations (14) and (15) gives equation (9) again, and the corresponding $k$ and $n$ are expressed as equations (16) and (17), respectively. Hence, corrosion kinetics with either homogeneous nucleation or heterogeneous nucleation obeys our C-M equation.

It is necessary to indicate that overlapping among pits on the dimension of the metal surface has not been taken into consideration in derivation of corrosion volume and corrosion mass in both cases of homogeneous nucleation and heterogeneous nucleation. Such an assumption could be regarded as compensated by another assumption of the power relation of the average pitting depth with corrosion time. In any case, the C-M equation stands, given a power relation of corrosion volume or mass as a function of corrosion time.

According to our corrosion experiments, the iron corrosion proceeded like a homogeneous nucleation. It seems also helpful to indicate that the concrete expression forms of the $k$ and $n$ in the C-M equation (equation 9) like equations (12) and (13) for an ideal homogenous nucleation, equations (16) and (17) for an ideal heterogenous nucleation, or their mixing case do not influence the application of our new and unified inhomogeneity parameter $M_{\mathrm{corro50\%}}$, namely, corrosion mass at 50% coverage simply called half-coverage mass.

## Supplementary Figures and Tables

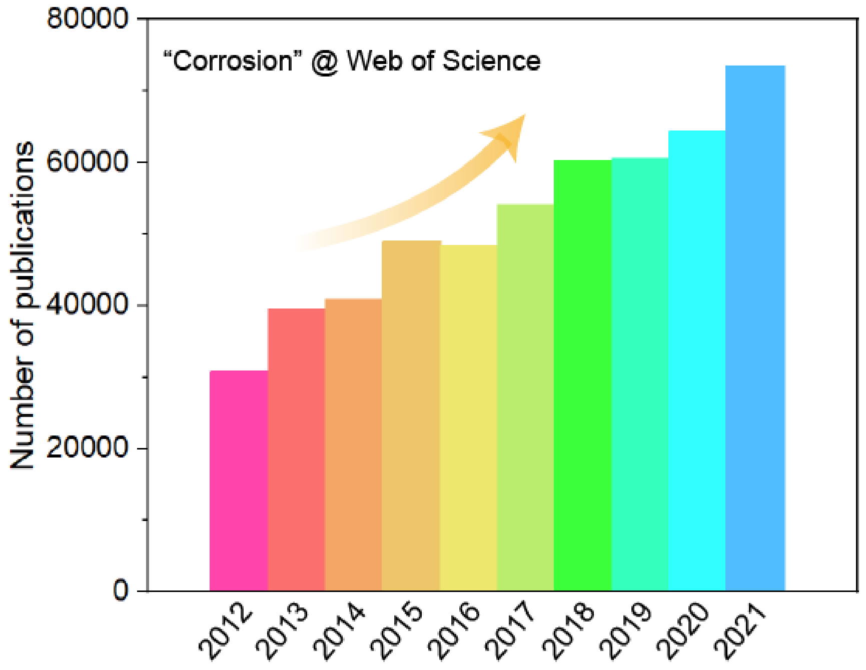


**Figure S1.** Publication statistics about "corrosion" during the latest 10 years.

**Source**: Web of Science.

**Subject**: Corrosion

**Results**: There are about **580,000** publications during the latest 10 years, including **1065** "highly cited papers" and **35** "hot papers". Annual publications are shown in the histogram, where 2022 result is incomplete now and thus not shown.

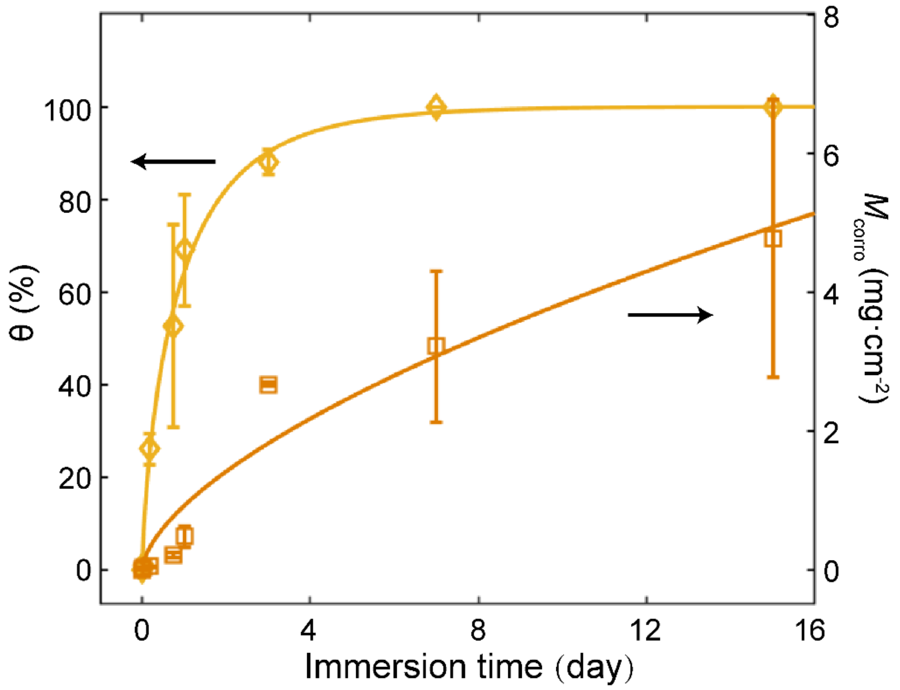


**Figure S2.** Experimental data of corrosion coverage (θ) and corrosion mass ($M_{corro}$) of iron sheets in deionized water (DI) and the confirmation of our theoretical prediction. The data of θ was fitted with equation (6) in the main manuscript $\theta = 1 - \exp\left(-k' t^{n'}\right)$ and the data of $M_{corro}$ was fitted with equation (8) $M_{corro} = at^{b}$. The fitted parameters $k$' and $n$' are 1.0 and 0.74, respectively; the fitted parameters $a$ and $b$ are 0.92 and 0.62, respectively.

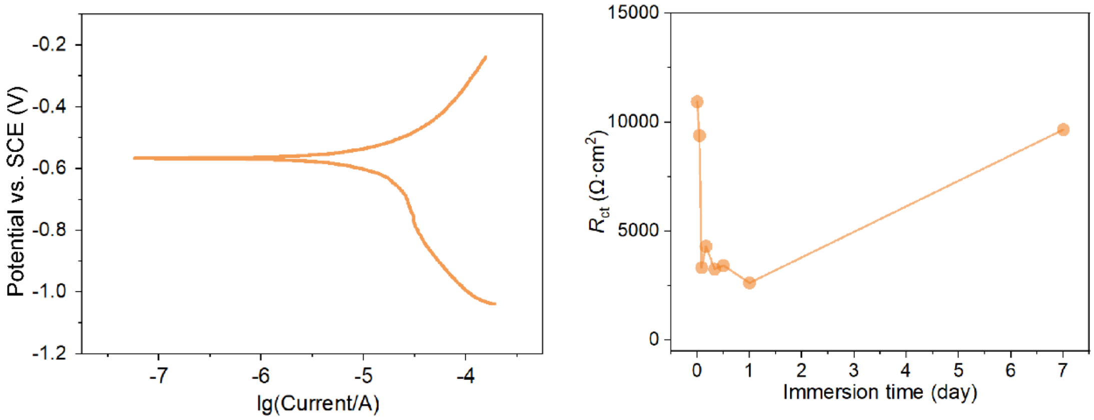


**Figure S3.** Results of an electrochemical measurement of iron corrosion in DI. Tafel curves of iron after immersed for 3 days (left), and charge transfer resistances $R_{ct}$ acquired by EIS for different immersion times (right).

**Table S1.** Ion and glucose concentrations (mol/L) of the aqueous corrosion media examined in this study.

| | $Na^+$ | $K^+$ | $Ca^{2+}$ | $Mg^{2+}$ | $Cl^-$ | $SO_4^{2-}$ | $HCO_3^-$/ $CO_3^{2-}$ | $H_2PO_4^-$/ $HPO_4^{2-}$ | Glucose |
|---|---|---|---|---|---|---|---|---|---|
| DI | - | - | - | - | - | - | - | - | - |
| AS | 0.48 | 0.0094 | 0.011 | 0.055 | 0.53 | 0.29 | 0.0024 | - | - |
| NS | 0.15 | - | - | - | 0.15 | - | - | - | - |
| PBS | 0.16 | - | - | - | 0.14 | - | - | 0.010 | - |
| HS | 0.14 | 0.054 | 0.0013 | 0.00090 | 0.19 | 0.00041 | 0.0042 | 0.00097 | 0.0056 |

DI: Deionized water; AS: artificial seawater; NS: normal saline; PBS: phosphate buffer saline; HS: Hank's solution.

**Table S2.** Fitted parameters of corrosion coverage (θ) versus time with equation (6).

| | DI | AS | NS | PBS | HS |
|---|---|---|---|---|---|
| *k'* | 1.0 | 2.2 | 0.44 | 0.015 | 0.0043 |
| *n'* | 0.74 | 2.2 | 0.87 | 0.72 | 0.33 |

**Table S3.** Fitted parameters of corrosion mass ($M_{corro}$) versus time with equation (8).

| | DI | AS | NS | PBS | HS |
|---|---|---|---|---|---|
| *a* | 0.92 | 0.13 | 0.63 | 0.32 | 0.12 |
| *b* | 0.62 | 1.4 | 1.2 | 0.58 | 0.96 |

**Table S4.** Fitted parameters of *k* and *n* by C-M equation of iron corrosion in different media.

| | DI | AS | NS | PBS | HS |
|---|---|---|---|---|---|
| *k* | 2.0 | 7.5 | 0.48 | 0.062 | 0.0090 |
| *n* | 0.72 | 1.9 | 0.86 | 1.2 | 0.39 |

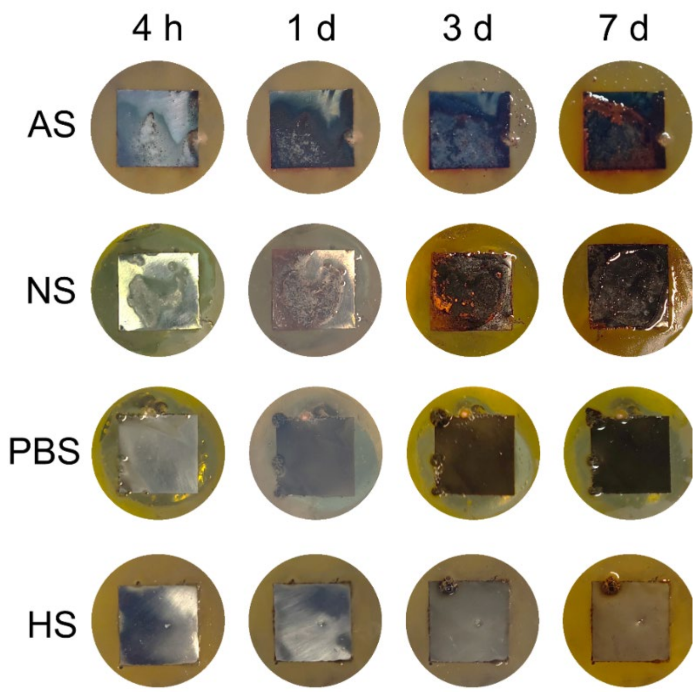


**Figure S4.** Global views of corrosion surfaces of iron sheets after immersion in the other four aqueous media, artificial seawater (AS), normal saline (NS), phosphate buffer saline (PBS), and Hank's solution (HS).

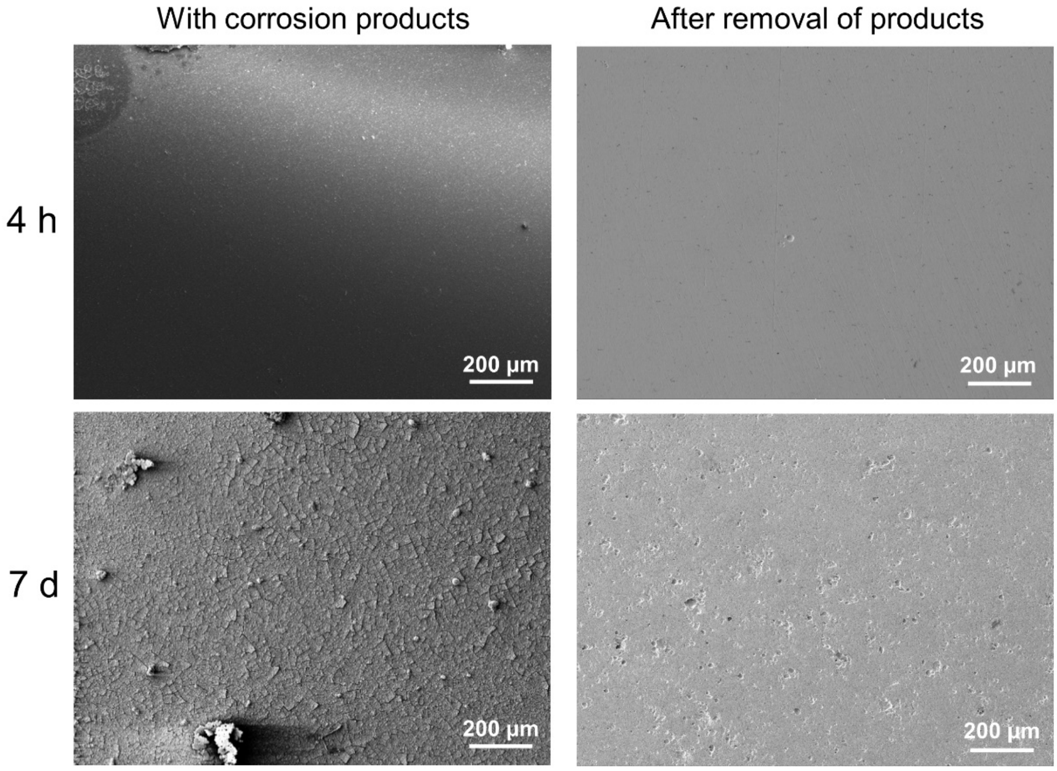


**Figure S5.** SEM images of iron surfaces after immersion in PBS for 4 hours and 7 days.

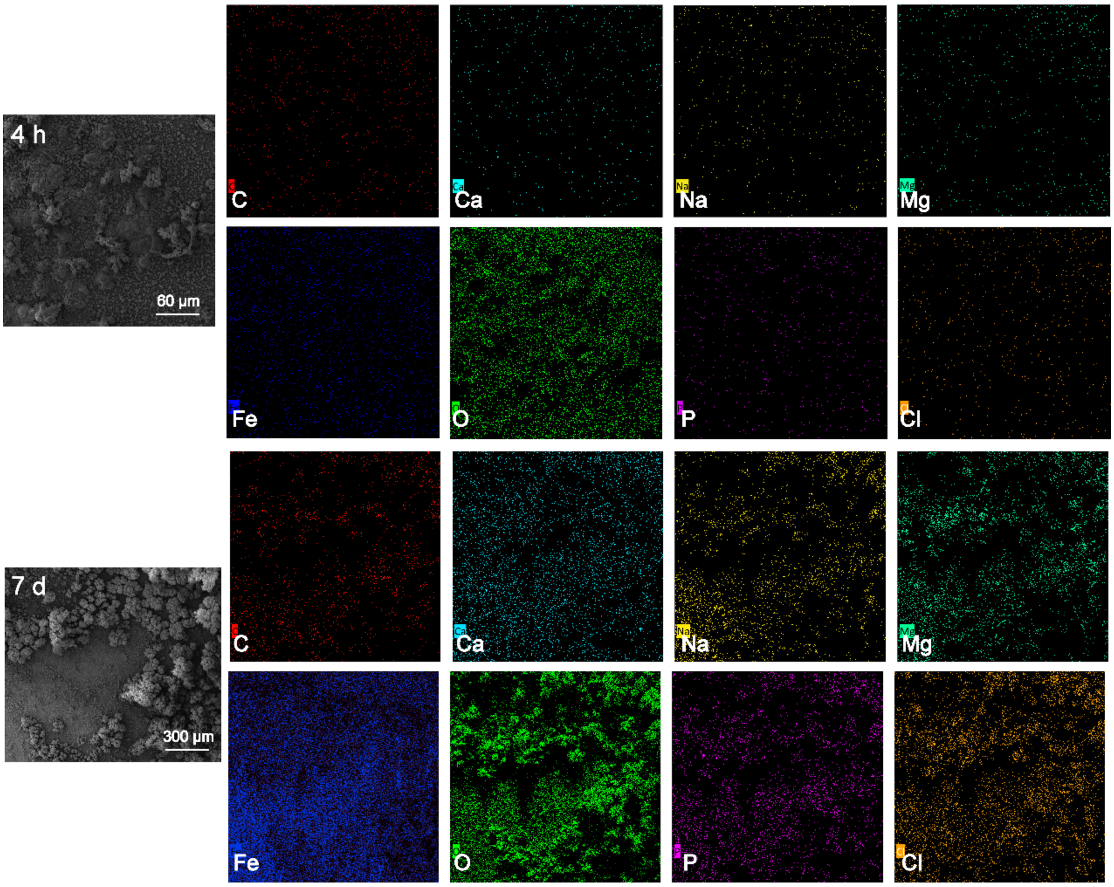


**Figure S6.** SEM-EDS images to show element distribution of iron surfaces after immersion in AS for the indicated time. The left is a normal SEM image, and the others are corresponding EDS images.

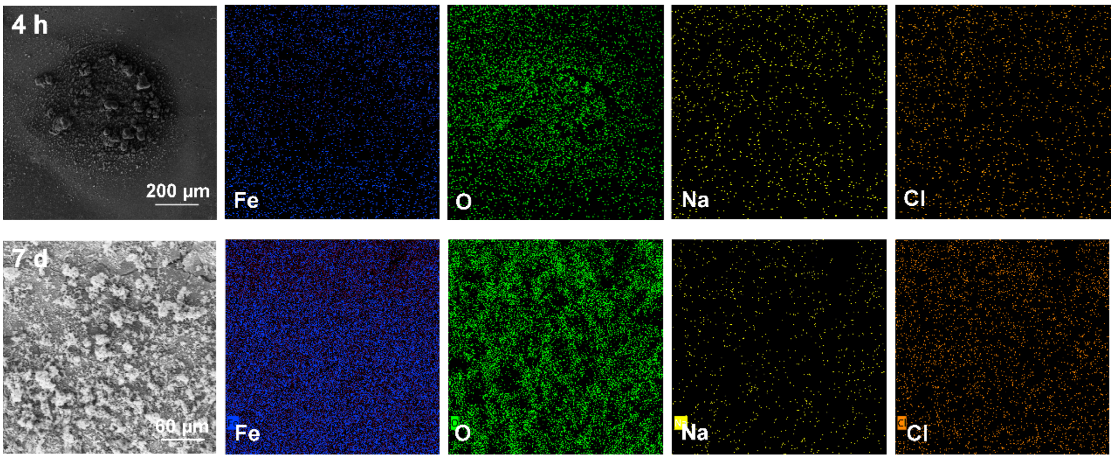


**Figure S7.** SEM-EDS images to show element distribution of iron surfaces after immersion in NS for the indicated time.

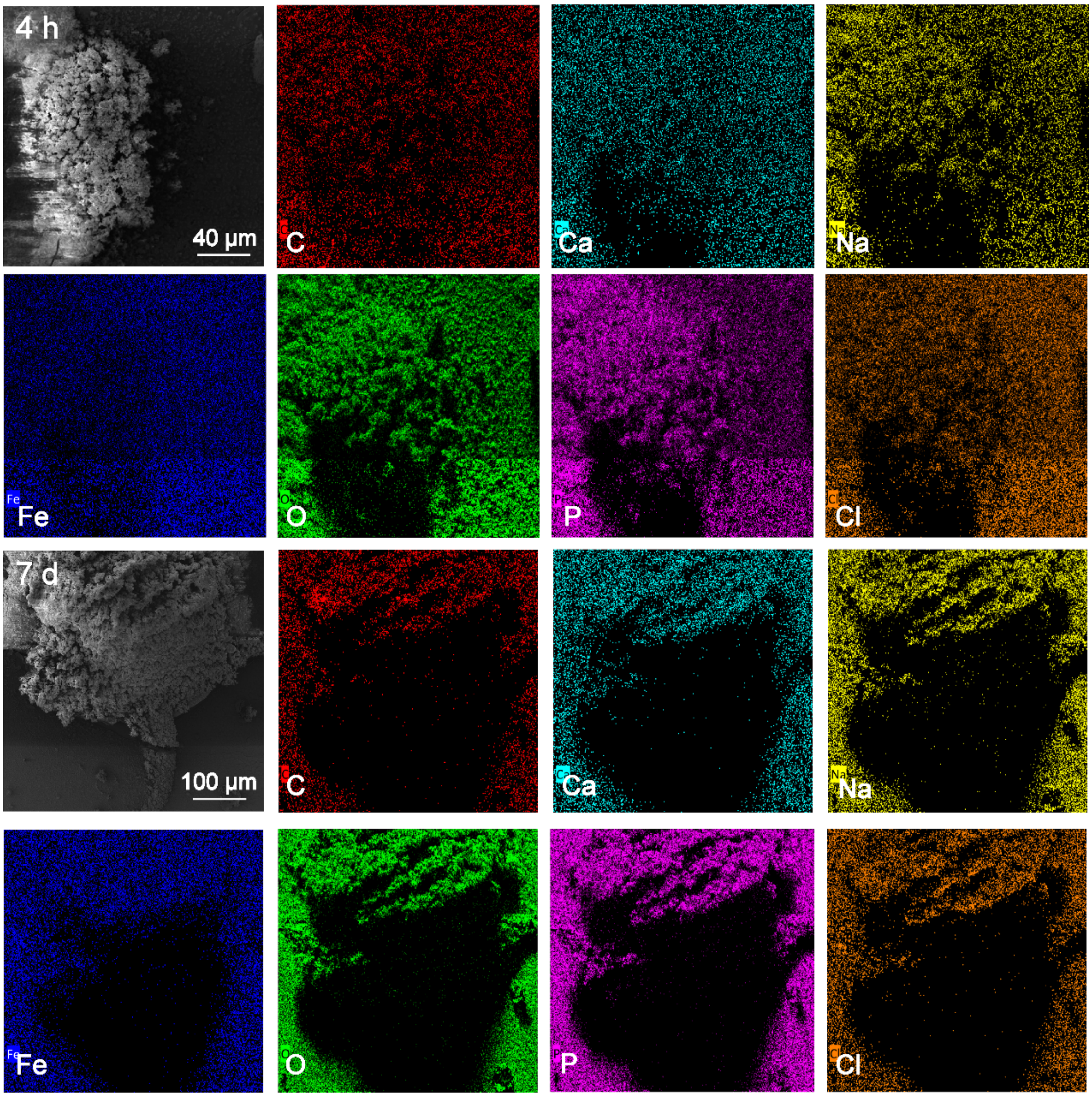


**Figure S8.** SEM-EDS images to show element distribution of iron surfaces after immersion in PBS for the indicated time.

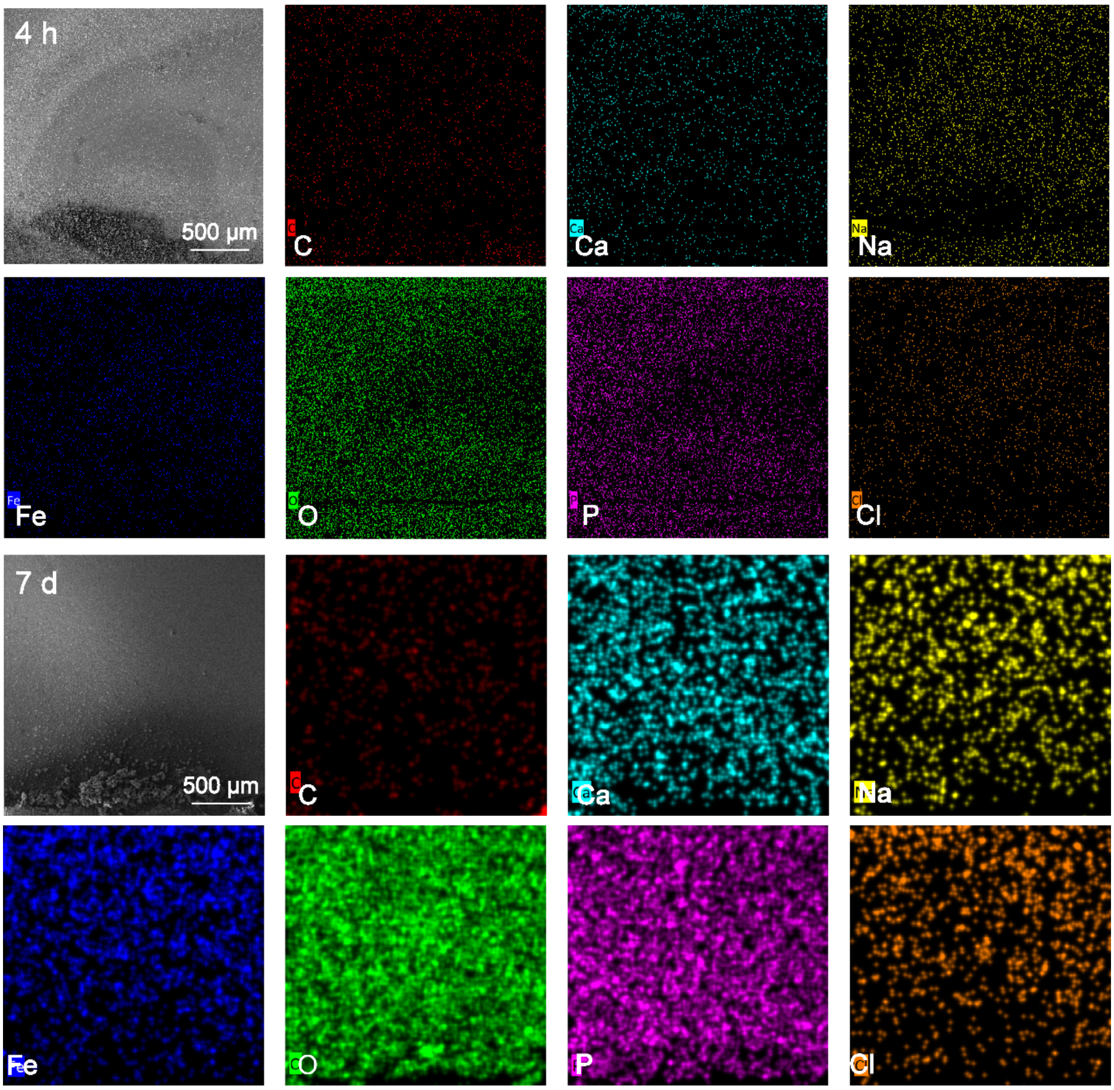


**Figure S9.** SEM-EDS images to show element distribution of iron surfaces after immersion in HS for the indicated time.

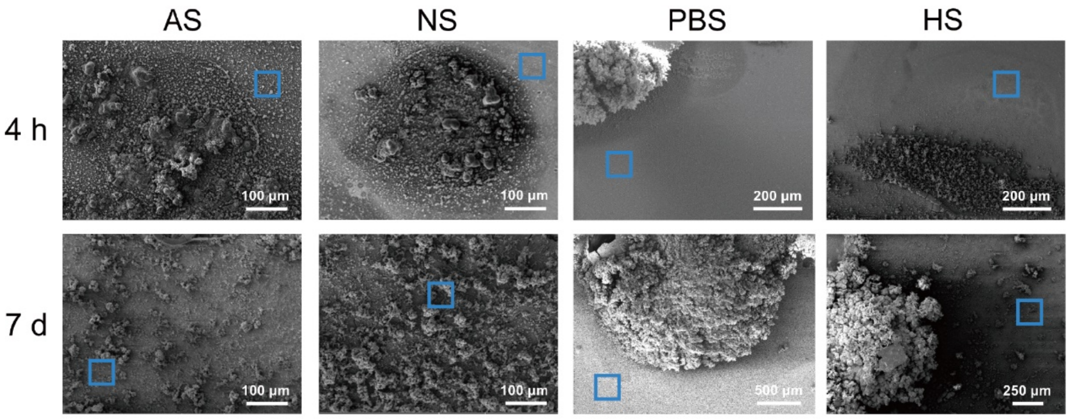


**Figure S10.** SEM images of iron after immersion for 4 hours and 7 days in the indicated four media. On the iron surface after immersed in AS for 7 days, the loose corrosion products on the specimen dropped off when the specimen was taken out of the medium.

**Table S5.** Element contents in the blue boxes of Figure S10 detected by EDS.

| | C | O | Fe | Ca | P | Na | Cl | Mg |
|---|---|---|---|---|---|---|---|---|
| AS-4 h | 1.3 | 18.6 | 73.4 | - | - | - | - | 6.7 |
| AS-7 d | - | 17.0 | 76.8 | - | - | 1.2 | 0.5 | 4.5 |
| NS-4 h | - | 13.6 | 86.4 | - | - | - | - | - |
| NS-7 d | - | 38.1 | 61.7 | - | - | - | 0.2 | - |
| PBS-4 h | - | 40.6 | 39.5 | 0.9 | 15.4 | 2.7 | 0.9 | - |
| PBS-7 d | - | 46.5 | 42.1 | - | 5.6 | 4.0 | 1.8 | - |
| HS-4 h | - | 30.4 | 58.6 | 3.0 | 3.7 | 2.9 | 1.4 | - |
| HS-7 d | 5.7 | 41.7 | 39.7 | 5.7 | 5.6 | 1.6 | - | - |

Note: The uncorroded area is selected to detect the ion deposition and passive layer formation if the coverage is low. If the corrosion coverage was, according to the global view, close to 100%, the corroded area was selected in the SEM images, to detect the element content.

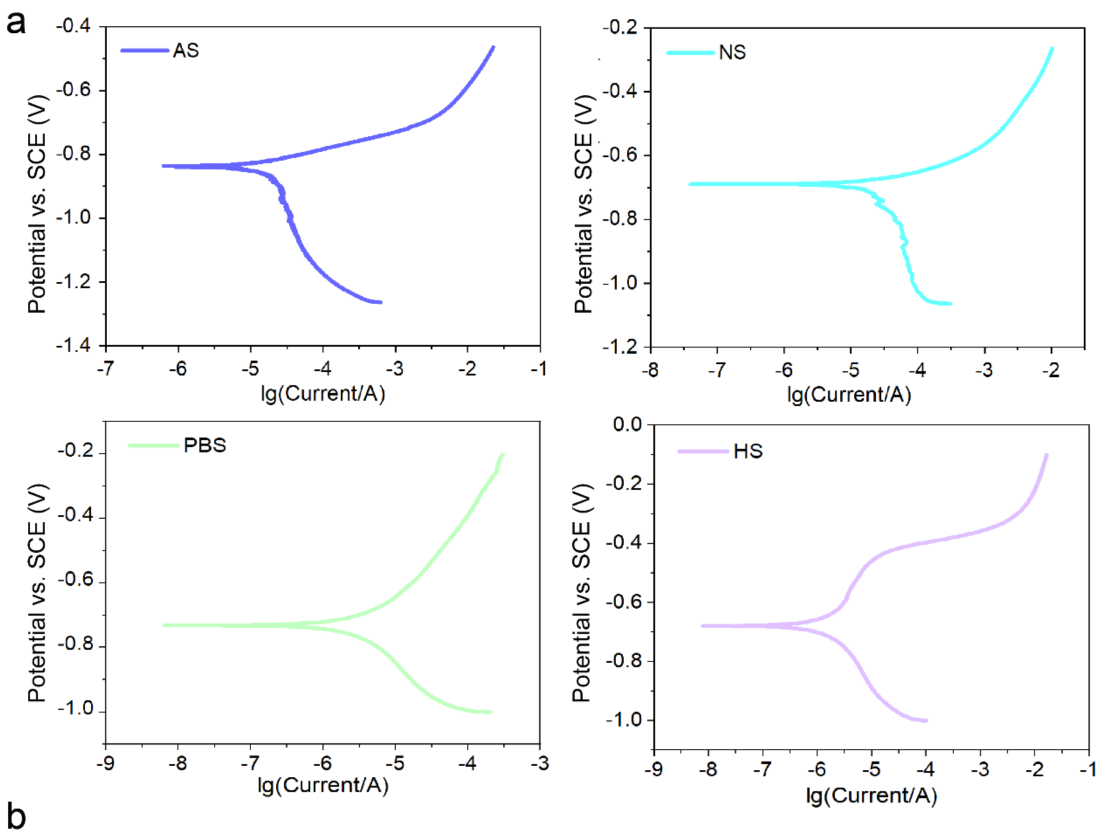


b

| | DI | AS | NS | PBS | HS |
|---|---|---|---|---|---|
| OCP (V) | -0.64 | -0.75 | -0.66 | -0.63 | -0.58 |
| Corrosion potential (V) | -0.57 | -0.79 | -0.69 | -0.73 | -0.68 |
| Corrosion current (μA·cm$^{-2}$) | 10.1 | 27.9 | 23.7 | 3.5 | 2.5 |

**Figure S11.** Potentiodynamic tests of iron in different media. (a) Tafel curves of iron after immersion for 3 days. A saturated calomel electrode (SCE) was set as the reference electrode. (b) Calculated open circuit potentials (OCPs), corrosion currents, and corrosion potentials of iron sheets in different media.

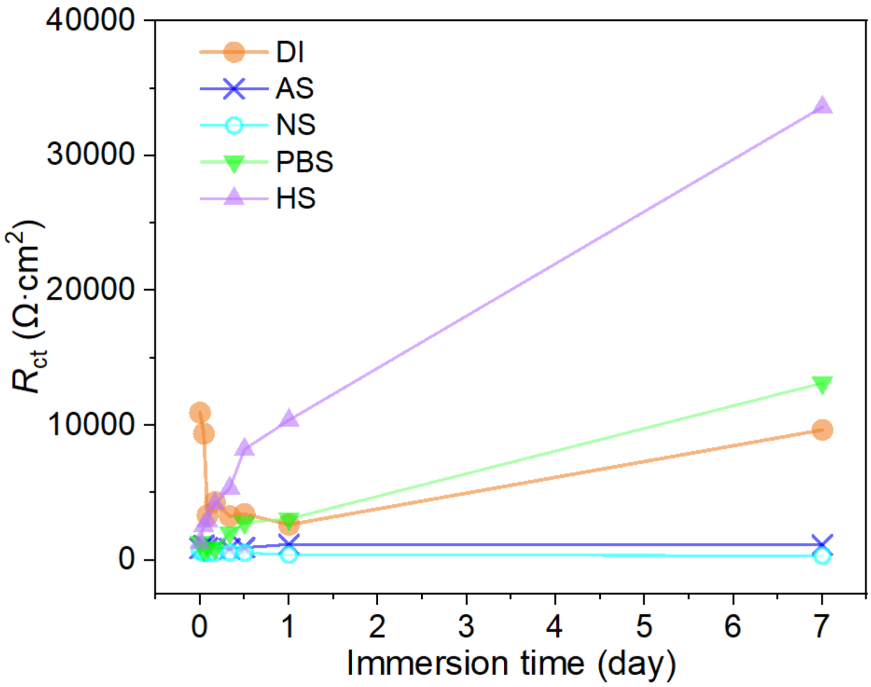


**Figure S12.** Charge transfer resistance $R_{ct}$ of iron after immersion in the indicated 5 different media.

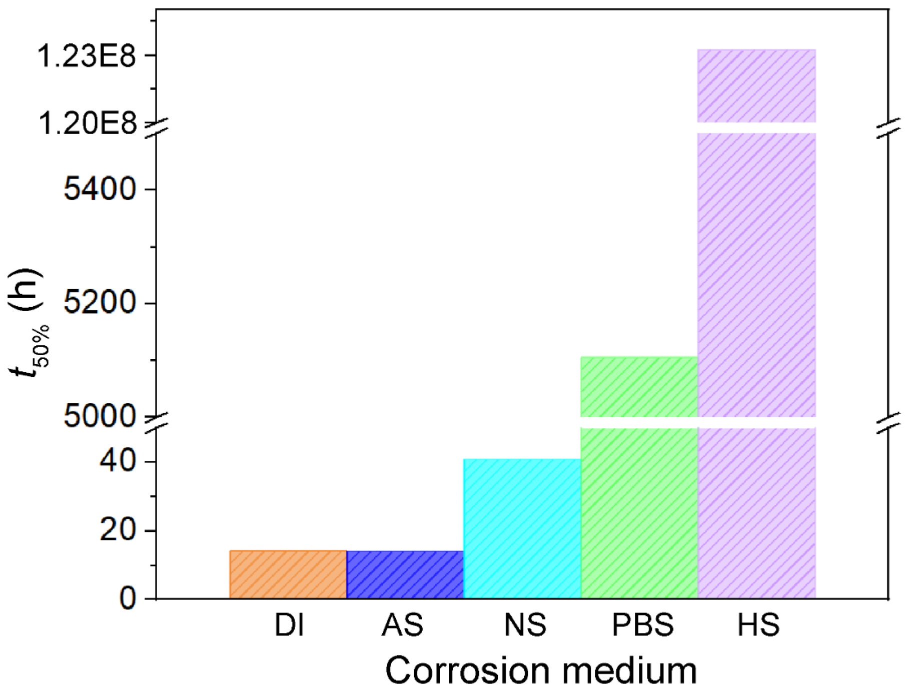


**Figure S13.** The calculated values of time with respect to 50% corrosion coverage of iron after immersion in the five media.

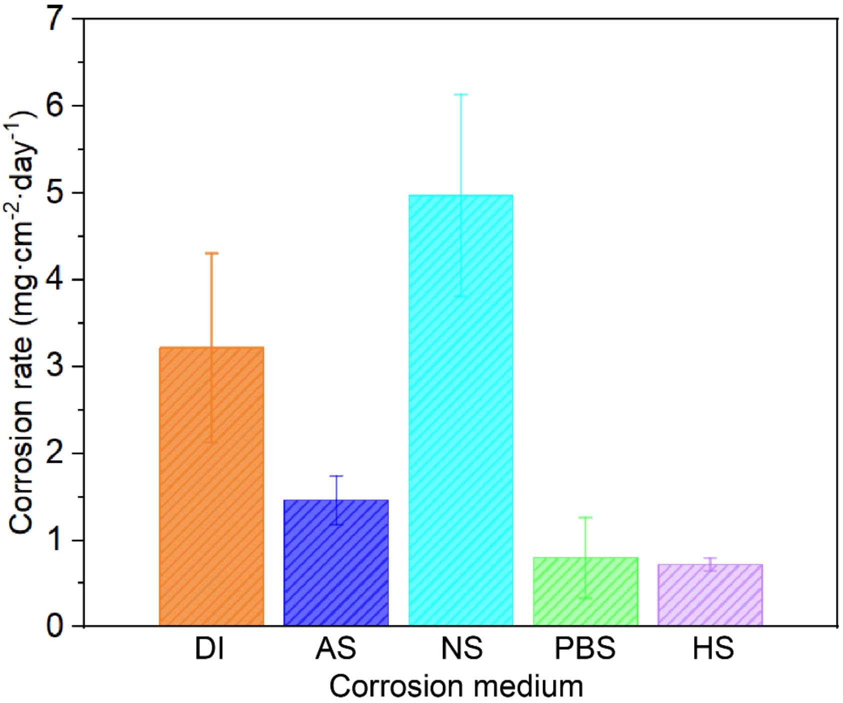


**Figure S14.** The corrosion rates calculated based on corrosion mass within 7 days.
